# Factors associated with lost to follow-up (LTFU) among patients with hypertension: A scoping review

**DOI:** 10.1371/journal.pgph.0006240

**Published:** 2026-06-30

**Authors:** Raksha Kamath, Weena Stanley, Partha Protim Hazarika, Prajwal Lemuel Salins, Bhageerathy Reshmi

**Affiliations:** 1 Department of Health Information Management, Manipal College of Health Professions, Manipal Academy of Higher Education, Manipal, India; 2 Department of Medicine, Kasturba Medical College, Manipal Academy of Higher Education, Manipal, India; 3 Department of Health Informatics, MGM School of Biomedical Sciences, MGM Institute of Health Sciences (MGMIHS), Navi Mumbai, India; Indiana University South Bend, UNITED STATES OF AMERICA

## Abstract

Regular follow-up appointments are essential for effective hypertension management; however, many patients fail to attend scheduled visits and become lost to follow-up (LTFU). Understanding the factors associated with LTFU is important for preventing hypertension-related complications such as stroke, cardiovascular disease, and kidney disorders. This scoping review aimed to identify factors associated with LTFU among patients with hypertension and to summarize the terminology and timeframes used to define LTFU in the literature. A scoping review was conducted following PRISMA-ScR guidelines. A systematic search was performed for studies published between 2010 and 2025 across MEDLINE, Scopus, Ovid MEDLINE, Web of Science, Google Scholar, and grey literature sources. The review protocol was registered with the Open Science Framework (OSF) (Registration: https://osf.io/a2wsg/). The methodological quality of included studies was assessed using design-specific tools. A total of 4,039 records were identified, and after removing duplicates, 2,956 articles were screened. Thirteen studies met the inclusion criteria, including six cross-sectional studies, four mixed-methods studies, one qualitative study, one retrospective case–control study, and one cohort study. Nine studies were rated as high quality and four as moderate quality. The definitions and terminology used to characterize LTFU varied considerably across studies. Factors associated with LTFU were grouped into five categories: patient-related, treatment and disease-related, healthcare provider-related, health service and system-related, and interpersonal factors. Patient-related factors were most frequently reported, followed by treatment and disease characteristics, while healthcare provider, service, and system-related factors were less commonly described. Interpersonal factors were reported in only three studies. This scoping review identified a range of patient-, treatment-, healthcare provider-, and health system–related factors contributing to LTFU among patients with hypertension. The findings highlight the complexity of LTFU in hypertension care and underscore the need for targeted strategies to strengthen follow-up mechanisms, improve patient engagement, and enhance continuity of care.

## Introduction

Patients missing their clinical appointments is a typical occurrence in the delivery of healthcare worldwide and for a variety of diseases [1]. Missed appointments undermine the continuity and effectiveness of healthcare delivery, interrupt routine monitoring of health status, and may increase healthcare costs [1,2]. Patients often fail to follow up on medical advice due to age, illness severity, illiteracy, lack of awareness, and financial burden [3,4] thereby, compromising effective management and quality of life [5]. Terminating therapy is a concern, linked with negative outcomes of health requiring immediate re- intervention [6].

In this context, patients who become lost to follow-up (LTFU) represent an important challenge for healthcare systems. LTFU occurs when patients discontinue scheduled follow-up visits and their subsequent clinical outcomes become unknown [7–9]. Inadequate follow-up can lead to disease progression, increased morbidity, and mortality, affecting patient care and faith in the healthcare system [3,4,10]. Individuals with more serious conditions are more likely to consult multiple physicians and to experience poorer outcomes [11].

Although chronic illnesses necessitate routine monitoring, frequent loss to follow-up might make long-term follow-up difficult. Failure to follow treatment leads to worse illness management, hospital admissions, and higher death rates. LTFU is a public health concern in high-income countries [4]. Patients who are LTFU impose higher demands on primary services than those who will be completing the treatment regimen. Costs on hypertension increases, the burden will shift to the patients leading to treatment attrition and financial catastrophe [12].

Hypertension is a common but treatable public health concern globally [13], mainly due to its high prevalence and its association with cardiovascular and renal disorders [14,15]. It is a leading global cause of untimely death. Non-communicable diseases (NCDs) are the primary cause of death and morbidity worldwide [16–18]. 9 million deaths reported worldwide are attributed to hypertension annually, leading to significant public health concern [19]. An estimated 1.28 billion people between the ages of 30 and 79 globally suffer from hypertension; two-thirds of these individuals reside in low- and middle-income nations. Of them, 46% of adults are unaware that they have the disease [20–22]. It is estimated that 7.5 million deaths globally, or about 12.8% of all deaths [23,24] are caused by systolic hypertension alone. That amounts to 57 million (3.7%) disability-adjusted life years (DALYs) [25,26]. LMICs (Low- and middle-income countries) face a high burden, with two-thirds of hypertension patients needing more medical attention [27].

To enhance the standard of care and support patients for their well-being, it is imperative to determine the characteristics linked to LTFU [4]. Reviews examining the risk variables for LTFU in patients with hypertension are limited. Prior research mostly looked on the variables related to missing appointments, medication compliance, adherence and non- adherence to therapy, and among the communicable diseases. Considering the rising concern over non-communicable illnesses and the fact that hypertension is one of the leading causes of illness and death, it is essential to investigate the risk factors for LTFU in individuals with hypertension.

Additionally, the idea of LTFU has been widely used in clinical settings to find patients who might have stopped receiving medical care. There isn’t a common definition of LTFU for medical appointments in the literature currently in publication. Accordingly, it is critical to clarify and understand the meaning of LTFU in the healthcare context, which benefits patients, healthcare professionals, and researchers and plays a crucial role from a clinical and research standpoint.

Therefore, it would be essential to get an overview of the words and concepts used to characterize LTFU [4,28,29]. Scoping research was conducted in order to provide a comprehensive understanding of the factors linked to LTFU among individuals with hypertensive illness. The primary objectives of the review were to identify the contributing elements or causes of LTFU in hypertensive individuals and to provide an outline of the terminology and definitions used to characterize LTFU in the included research.

## Methods

The Joanna Briggs Institute (JBI) methodology, which is informed by the methodological framework originally proposed by Arksey and O’Malley [30] and the reporting recommendations made in the PRISMA Extension for Scoping Reviews [31] were all utilized in this scoping review. This scoping review was conducted as per PRISMA-ScR and review protocol was submitted to the Open Science Framework and published under the following registration https://osf.io/a2wsg/

**Defining the research query:** The primary question prompted this review is; What are the factors associated with LTFU among hypertensive patients in accessing and adhering to the treatment?

**Identifying relevant studies:** An extensive literature search was conducted across electronic database search was conducted using PubMed, Ovid Medline, Scopus, Web of Science, google scholar and grey literature sources including organizational reports, conference proceedings, and government publications in accordance with the PRISMA-P reporting checklist. The reference lists of the relevant articles were used to conduct manual searches. Search strategy, study selection, quality, data extraction, and data analysis/synthesis were all steps in the process. The databases were searched from January 2010 to December 2025, and the databases were last searched on 9 December 2025. The start date of January 2010 was selected to capture contemporary evidence indicating recent advancements in hypertension management and healthcare delivery systems. Only English-language papers published between 2010 and 2025 were included in the search due to feasibility constraints related to translation. Studies from all geographical regions were considered, with no restrictions based on country or setting. Keywords used were “Hypertension OR hypertensive OR hyperten* OR high blood pressure* OR elevated blood pressure* OR abnormal blood pressure* AND Lost to follow up OR No-show patient* OR Patient dropout* OR missed visit*” (Supplementary material: S1 File (Search Strategy))

**Selection of the eligible studies:** Studies were selected if reported factors associated with LTFU among patients with hypertension or explored reasons cited by patients or healthcare providers for missed follow-up or discontinuation of care and being LTFU for treatment. The evaluation excluded - reviews, meta-analyses, case reports, case series, studies that focused on other illness variables and causes, research published prior to 2010. For the purpose of this review, lost to follow-up (LTFU) was defined as patients with hypertension who did not attend scheduled follow-up visits within a specified time frame, as reported in the included studies. Additionally, as this scoping review aimed to capture variations in terminology, terms such as missed appointments, non-attendance, treatment gaps, and defaulters were extracted from the literature and considered where they conceptually aligned with LTFU within the context of each study. Hypertension was operationally defined as systolic blood pressure ≥140 mmHg and/or diastolic blood pressure ≥90 mmHg, or as defined by the included studies.

Following removal of duplicates, titles and abstracts were screened for relevance. Studies were excluded at this stage if they were not related to hypertension, did not address LTFU, or did not meet the inclusion criteria. Two reviewers (RK, BR) independently screened the full text of all pertinent articles using the inclusion and exclusion criteria after screening the titles and abstracts. Discussions and consensus were used to settle disagreements about the choice of articles.

### Data extraction

Data charting: A data extraction form was developed to extract information such as the author(s), year of publication, study location, study design, study setting, study population and disease studied, study aim(s) & objective(s), age, the percentage of patients who were LTFU, the median age of being LTFU for treatment, the main outcome, factors related to LTFU, LTFU definition, the source of LTFU, or how LTFU was identified. The respective authors were emailed when additional information was needed for specific articles. When the authors did not respond, the reviewers discussed about the ambiguities until they came to an agreement. The data charting table detailing study characteristics and extracted variables has been submitted as a supplementary material, S1 Table (Data charting table).

**Collating, summarizing, of results:** The findings were summarized using a narrative synthesis approach. Four categories—patient, healthcare system, service and provider, treatment and disease, and interpersonal characteristics—were inferred from the data. The percentage of hypertensive patients who were LTFU, the average age at which LTFU occurred, the source used to identify LTFU, and the criteria and terminology used in the included studies were all collected and compiled.

**Data analysis and reporting:** To report the search, a PRISMA-ScR flow diagram was utilized. The decision-making process, search results, duplicate citation removal, study selection, complete retrieval, further bibliography extraction, and final summary presentation are all shown in the flow diagram.

**Quality Appraisement:** The quality of the studies was assessed using the Newcastle-Ottawa quality assessment scales (NOS) for case control studies, the MMAT mixed method evaluation tool, and the Joanna Briggs Institute (JBI) quality rating standards [32–35]. Using the relevant checklists according to each study’s design (cohort, cross-sectional, case control, mixed-method, and qualitative), the methodological elements of each study were evaluated. The goal of the quality appraisal was not to weed out the studies, but to find and evaluate the benefits and drawbacks of the methodology used in the included studies. A “zero” was assigned to a study if a component of a checklist item was not reported or included, a “two” for reporting it, and a “one” for being unclear. The total of these ratings was then used to determine each study’s overall score. The quality level for each individual study was determined by dividing the total score (numerator) by the total potential score (denominator). On the JBI and MMAT checklists, study papers with scores of ≥66.7%, 33.4%–66.6%, or ≤33.3%, respectively, were categorized as high, medium, or low quality. Similarly, studies that received NOS scores were categorized as high, moderate, or poor quality according to how many stars they received for meeting the high-quality criteria [32–35].

## Results

### PRISMA

**Fig 1:**
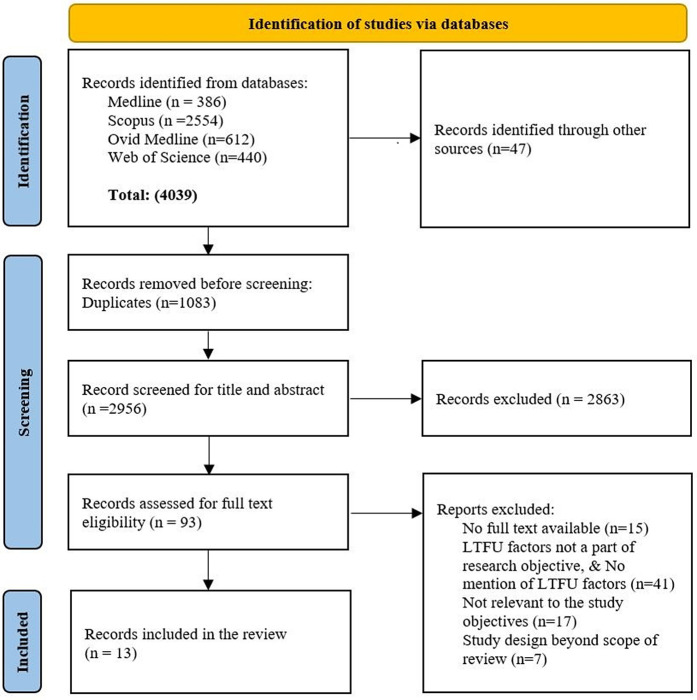
Study selection process flow diagram for preferred reporting items for systematic reviews and meta-analyses. This figure illustrates the identification, screening, eligibility, and inclusion of studies in the scoping review.

Steps involved in identifying and incorporating the studies into the review are shown in Fig 1. A total of 4,039 records—including 386 Medline, 2554 Scopus, 612 Ovid Medline, and 440 Web of Science articles—were obtained from the database search. There were 47 records found using other sources. 1083- duplicate records were eliminated, and 2,956 records were reviewed. After the title and abstracts were examined, 93 records subjected to full text screening. 13 articles, as shown in the PRISMA flow chart, were included in the review.

Of the 13 included studies represented diverse methodological approaches, including cross-sectional, mixed-methods, qualitative, cohort, and case–control designs. These studies were conducted across varied geographical settings, including low-, middle-, and high-income countries, reflecting diverse healthcare systems and patient populations. The study populations primarily consisted of adult patients diagnosed with hypertension, with variations in demographic and clinical characteristics. Sample sizes, healthcare settings, and approaches to defining and identifying LTFU varied considerably across studies.

### Overview of the included articles

This review included a different study design, such as mixed, qualitative, and quantitative, among which case control (1) [36], Mixed method (4) [1,16,37,38], cross- sectional (6) [39–44], Qualitative (1) [26], cohort(1) [45]. The chosen studies were carried out in a different country, including South Africa (1) [1], Nigeria (1) [41], Ethiopia (3) [40,42,44], India (2) [26,37], Oman (1) [6], Sierra Leone (1) [16], Nepal (1) [38], Lebanon (1) [43], Switzerland (1) [45], and Pakistan (1) [39]. Table 1 lists the study features of the included papers, including the nation, study design, care setting, study objective, sample size.

**Table 1:** Attributes of the included articles.

STUDY, YEAR, LOCATION	STUDY DESIGN	STUDY SETTING, SAMPLE POPULATION	OUTCOME	PATIENTS LTFU(%)	MEDIAN AGE OF BEING LTFU	LTFU DEFINITION	SOURCE OF MISSED APPOINTMENTS/ LTFU	FACTORS ASSOCIATED WITH LTFU			
								Patient characteristics	Healthcare system, service & provider characteristics	Treatment & Disease related characteristics	Interpersonal factors
Alawadhi. A, 2023, Oman	Retrospective case–control study	Tertiary Referral Hospital, N = 343149	Missed apointments/ Visits (Pre and Post Covid)	LTFU: PreCovid 19 Pandemic:30156 (16.9%) NonLTFU:Pre Covid 19 Pandemic: 148693 (83.1%) LTFU: Post Covid 19 Pandemic:30896 (23.8%) Non LTFU Post Covid Pandemic: 98788 (76.2%) Virtual Appointment during Covid 19 pandemic: LTFU: 7,447(32.2%)	31-40 years: PreCovid 19 Pandemic: 40200 (22.5), Post Covid 19 Pandemic: 30017 (23.1)	A missed appointment was defined as a patient who had a scheduled appointment but did not attend the appointment without contacting the hospital to cancel or rebook the appointment. This was recorded in the system as failed to attend.	Electronic Health Records	v	v		
Dibba. Y, Kono; Sierra Leone, 2024	Convergent mixed-methods study.	Secondary Level Hospital, N = 1656	(i)Adherence to the treatment protocol ii) loss-to- follow-up (LTFU) iii) Engaged in but not fully adherent	22%	40-60 years	**LTFU**: Loss-to- follow- up (LTFU) was defined as consecutively missing appointments for 6 months. **NON- ADHERENCE: Patients who attended <80% of clinic appointments but not LTFU were considered engaged in the clinic but not adherent to the prescribed regimen of care.**	Electronic Medical Records, Interviews	v	v	v	
Y. Mamo, Ethiopia, 2019	Cross-sectional survey	Five health centres NCD Clinics, N = 135	Lost to follow up	n= 37(27.4%)	18-54 years	Clinic LTFU was defined as non-attendance from follow-up of 6 months	“Interviews (Questionnaires) “	v	v	v	
Das. B, Punjab, 2020	Qualitative study: Telephonic interview	Public health facility, N = 300	Missed appointments are indicative of loss to follow-up	n=157 (52.3%)	<50 years	Patients who had missed their appointments for more than three consecutive months.	Medical Records & Telephonic interview	v	v	v	v
Jaswal. N, India, 2024	Retrospective follow-up concurrent mixed-method study	Health facilities (CHC’s, PHC’s), N = 1169	Patient retention rates in hypertension treatment across Health care levels	80-90%	40-60 years	The patients who were registered under NPCDCS/IHCI and followed up until the previous quarter did not return for a follow-up visit consecutively for 3 months or more over the 1-year study period.	Focus group discussion & In- depth interviews	v	v	v	v
Akinniyi. AA, Nigeria, 2017	Descriptive cross- sectional study	Outpatient clinic, N = 300	Missed medical appointments	16.70%	DNS	Missing more than 3 out of 10 medical appointments	Questionnaires	v	v	v	
Magadzire. BP, South Africa, 2017	Exploratory, cross-sectional mixed-methods design	Five PHC facilities, N = 89	Missed appointments & indicative to lost to follow -up	67%	DNS	DNS	“Indepth interviews (To yeild HCP and Patients expiriences) & medical record reviews (MRR))In order to establish whether missed appointments were temporary or indicative of loss-to-follow-up) “	v		v	
Mahmood. S, Pakistan, 2020	Cross-sectional study	Primary, Secondary & tertiary healthcare settings, N = 662	Regularity in follow-up visits, Attendance to follow-up visits	388 (59%)	<60 years	Regularity in follow-up visits= If he/she had missed more than three out of 10 scheduled/advised follow-up visits	Questionnaires (Self- reported questionnaires, MMAS - 8 for assesing medication adherence, Questions/ Interviews to the patients), Patients Medical records	v		v	
S. Devkota et al., Kathmandu, Nepal, 2016	Cross-sectional mixed-methods study (quantitative + qualitative)	Municipalities of Kathmandu; Community based, N = 191	Awareness, Treatment, BP Control (hypertension care cascade)	Not directly reported (LTFU not quantified as %; only described qualitatively)	DNS	DNS (study discusses follow-up discontinuation but no formal LTFU definition used)	Self-report during interviews & FGDs (no administrative tracking)	v	v	v	v
Yirga GK et al., South Gondar Zone, Ethiopia, 2024	Institutional-based cross-sectional study	1 Comprehensive Specialized Hospital + 9 Primary Hospitals, N = 401	Adherent vs. Non-adherent appointment follow-up	39.2% non-adherent	58.47 years (median)	Missing >3 appointments out of 10 consecutive scheduled appointments	Self-report + chart review	v	v	v	v
Abbas H et.al, Lebanon,2020	Cross-sectional study	Community pharmacies (40.2%), tertiary hospital (33.3%), private clinics (26.5%), N = 1497	Adherence vs. non-adherence to antihypertensive medications	244 patients = 16.3% non-adherent	DNS	Non-adherence was defined based on patient self-report of not taking antihypertensive medications as prescribed by their physician. No specific time frame or percentage was provided.	Self-reported questionnaire administered via trained interviewers	v	v	v	v
Saguner A.M. et al., Bern, Switzerland, 2010	Prospective longitudinal study	Medical Outpatient Unit, University Hospital of Bern, N = 89	Hypertensive crisis (SBP ≥ 200 mmHg and/or DBP ≥ 120 mmHg with acute rise)	4.5% (4 patients lost to follow-up)	DNS	Patients who did not complete follow-up because they moved away or left the country	Medical record review + structured patient interviews + GP interviews	v	v	v	v
Belay DG & Adugna A, Northwest Ethiopia (Gondar City), 2022	Institution-based secondary data review	9 public health facilities (8 health centers + 1 specialized hospital), N = Total: 319,693 chronic disease patients: HIV/AIDS: 296,593, Hypertension: 12,744, Diabetes: 10,356			DNS	“Patients who did not take ART and/or antihypertensive and/or DM medications for at least 1 month or more were categorized as LTFU.”	(Demographic and Health Information System) DHIS-2 aggregated health-facility monthly reports	v	v	v	

**The sample size and study settings of the included papers:** Of the thirteen articles, three studies were conducted in tertiary referral or university hospital settings [6,41,45]. Two studies were carried out in secondary-level hospitals [16,26]. Four studies were undertaken in primary healthcare or community-based settings, including primary health centres, community health centres, rural health centres, and municipality-level community settings [1,37,38,40]. Few studies utilized mixed healthcare settings: one study included both primary and secondary healthcare facilities [44]; one spanned all three levels of care (primary, secondary, and tertiary) [39]; one was conducted across one comprehensive specialized hospital and nine primary hospitals [42]; one from multiple care points including community pharmacies, tertiary hospitals, and private clinics [43].

The range of sample sizes was 89 [1,45] – 343149 [6]. Ten studies included only adults with hypertensive disease [6,16,26,37–40,42,43,45] and three studies included mixed chronic disease populations in which two included diabetes and hypertensive populations [1,41] and another one included diabetes, hypertension and HIV/AIDS [44]. A variety of outcomes have been identified from the included studies, such as missed appointments, treatment protocol adherence, lost to follow up, missed appointment indication to LTFU, missed doctor’s appointments, patient retention, follow-up visit attendance, blood pressure control along the hypertension care cascade. adherence to medications and follow-up appointments; hypertensive crises; and increased lost-to-follow-up, particularly during COVID-19, along with reduced initiation of new hypertension treatments.

**Age in which the study was conducted:** In each study**,** the mean ages of the patients varied - ranging from 12 [16]– 64 [41] years old. Most of the studies which were included were carried out with young individuals and the elderly population.

**Source of lost to follow-up:** Across the included studies, LTFU was determined by various sources; including medical records review [1,6,16,26,39,42,45], by using In- depth interviews [1,16,37,38,42,43,45], focus group discussions [37,38], telephonic interviews [26], Questionnaires [39–41] and from aggregated DHIS-2 health-facility reports [44], with some articles using both medical records and interviews to determine LTFU.

**Quality of the Studies:** Nine of thirteen articles were of high quality [1,16,26,37,38,42–45], whereas the other four were of moderate qualit**y**[6,39–41].Common methodological problems were noted, including inadequate descriptions, a failure to identify confounding factors and strategies to address them, improper outcomes measured in a valid and reliable manner, and the mixed methods study did not specify the adequate rationale for using the study design.

### Terms of Missed appointments and lost to follow-up

The review’s included studies used a variety of terms to describe missing visits and LTFU (Table 1), including “Missed appointments” [1,6,26,41], “non- attendance” [40,41], “treatment gaps” [37], “missed medical appointments” [1,6,26,40,42], “appointment non- adherence” [41,43], “defaulters” [26], “lost to follow -up” [37,40,45], “loss to follow-up” [1,16,26].

### Definitions of Missed appointments and Lost-to follow-up

There was significant variation in the operational definition of LTFU for hypertension treatment. The majority of the research looked at follow-up and appointment status over a period of time [16,26,37,40,44]. Missed appointments were defined in studies as patients who had an appointment but failed to show up without calling the hospital to reschedule or cancel [6] and those who missed their appointments for more than three consecutive months [26], had missed more than three out of 10 scheduled/advised follow-up visits [39,42]. Missing more than 3 out of 10 medical appointments [41]. Additionally, patients who missed six consecutive appointments or who attended the clinic but did not complete the recommended course of treatment were considered lost to follow-up [16]. Non-attendance from follow-up of 6 months [40]. did not attend a follow-up appointment for three months or longer in a row during the one-year study period [37]. Non-adherence based on patient self-report of not taking antihypertensive medications as prescribed without specifying a time frame or percentage [43], patients who did not complete follow-up due to moving away or leaving the country [45], or patients who did not take ART, antihypertensive, or diabetes medications for at least one month [44].

The rate of LTFU for hypertension treatment ranged from 4.5% [44] - 90% [37]. The median age of being lost -to follow-up for hypertensive treatment ranged from 18 [40]– 60 [16,37,42] years, whereas five articles did not specify the median age of patients being LTFU for treatment [1,41,43–45]. To determine whether a patient was deemed LTFU or not, the studies that defined LTFU used a timeframe as a marker [16,37,39,40,44]. And even the studies that mentioned about missed appointments include the timeframe as a marker to indicate how the patient missed the appointments [6,26,41]. The duration ranges widely, from more than three months to twenty-four months. The duration of LTFU or missed appointments was chosen either in accordance with prior research studies or clinical norms.

### Factors associated with lost to follow-up for hypertensive treatment were classified as

Patient factors, healthcare system factors, service & provider factors, treatment and disease factors, and interpersonal variables are the categories used to describe the factors linked to LTFU for hypertension treatment. Table 2 lists the variables related to each study by all three categories and is classified by its results. Most of the studies considered factors from – Patient [1,6,16,26,37–45], Healthcare system, service & provider [6,16,26,37,38,40–42,44,45] and treatment & disease factors [1,16,26,37–45], and interpersonal factors [26,38,41–43,45] were examined from the studies.

1) **Patient Characteristics:** The number of studies looking at different patient characteristics from the respective studies is listed in Table 2. Numerous patient attributes were shown to be associated with lost-to-follow-up: Socio- demographics (age, sex (primarily women), older age groups, education (low levels), employment, socioeconomic status/income (low levels), insurance type, or health coverage (none), status of marriage, The distance between their place of living and the medical facility. The cost of transportation, the cost of prescription drugs, knowledge of the disease, due to time constraints and work responsibilities forgetting about the appointments, family support, strike or public holidays and Mobility or migration from the previous residence were the factors leading to LTFU. Socio demographic factors like age, sex and education level, cost of transport, treatment and medications, distance from the patient’s residence to healthcare facilities, and no insurance coverage where the most often looked elements: age was examined in eight articles, sex in four articles, and education level in three articles. Cost of travel, treatment and medication were addressed in three articles, five articles included distance from the patient’s residence to healthcare facilities, no insurance or lower coverage in three articles.
2) **Healthcare System, Service, & Provider Characteristics:** Ten articles addressed the characteristics of healthcare systems, services, and providers. longer wait times, service fees or costs, days of appointments (e.g., appointments at the start and end of the week were less likely to be missed), no appropriate education or awareness about the disease by the providers, Lacking in guidance and instructions from the facilities (e.g., inadequate dietary counseling, inappropriate advice regarding the dates of the follow-up visits), lack of consistent guidelines, Inadequate resources, insufficient medicine quality or availability, a poor rapport with the provider, inadequate follow-up, different doctors seen at each visit, medication stockouts and dissatisfaction with the standard of care received. Jaswal et.al found that, community health centers (CHC’s) had greater rate of patients being LTFU for treatment than being LTFU in lower healthcare facilities like Primary healthcare centers (PHC’s) and Health and Wellness clinics (HWC’s).
3) **Treatment & Disease related Characteristics:** Disease and treatment related characteristics were examined in twelve articles. Patients who had no blood pressure control (e.g., Blood pressure of >180/110mmHg) had greater rate of being LTFU. Absence of symptoms, Misunderstanding about the long-term management of the treatment, Failure to cure or no improvement found in disease, Side effects of drugs, Absence as well as presence of co-morbid conditions, lack of perceived illness, Duration of treatment (e.g., > 5 years of taking treatment for the disease condition), Pill burden (more number of medications prescribed), prescribed long term or lifelong medications, monotherapy, lack of lifestyle counseling, stopping follow up visits, uncontrolled blood pressure was strongly associated with non- adherence, poor medication adherence or compliance, too ill to attend the scheduled appointments, no perceived benefit received from the treatment, Preferring other modes of treatment (e.g., use of traditional medicines/ treatment) or switching to private medical care for further treatment. Number of medications/ pill burden, medication side effects, and being asymptomatic were the commonly examined factors.
4) **Interpersonal Characteristics:** Six of thirteen studies addressing interpersonal factors, i.e.,: negative attitude of healthcare provider, perceived disrespect towards the patients from the healthcare provider, lack of instructions and guidance from the healthcare facility or no proper communication to the patients or communication gaps with providers and patients, lack of reminder, social, emotional and psychological stress and poor relationship with healthcare providers were associated with LTFU for treatment.

**Table 2:** An overview of the four types of attributes that each study looked at.

Study, Year	Patient Characteristics	Healthcare system, service & provider characteristics	Treatment & Disease related characteristics	Interpersonal factors
Alawadhi. A et.al,2023 [6]	Sociodemographic: Sex, Age category, Nationality, Marital status, Type of Health insurance coverage, Distance from Patients residence to hospital.	Service cost, Longer waiting times, and Appointment days.	DNS (Did not specify)	DNS
Dibba. Y et.al, 2024 [16]	Age, Gender (Females), Health insurance, Transportation cost, Free medications/ Drug costs.	Long wait times, No frequent health education sessions/ talks.	Patients with blood pressure >180/110mmHg, Asymptomatic/ Absence of symptoms, Misunderstanding the long- term management of hypertension.	DNS
Y. Mamo et.al,2019 [40]	Age, Education level (illiterate), Socioeconomic status (low income level), Lack of knowledge, Cost of travel (by public transport/ walk), Unable to stop working, Distance from home to healthcare, Drug costs, Treatment costs.	Service dissatisfaction, Drugs not available.	Much improved health (Absence of symptoms), No improvement in disease, Failure to cure, Drug side effects, Preferring other modes of treatment (use of traditional medicines/ treatment), Misunderstood about the treatment, Did not feel unwell during scheduled appointment/Asymptomatic	DNS
Das. B et.al, 2020 [26]	Far distance from the facility, No time for a visit.	Lack of instructions/guidance from the facility, No proper instructions on when and where to come for a follow-up visits, long waiting time at the facilities, Poor quality of medications at public facilities, Medicines unavailability.	Lack of perceived illness, Acute health conditions/disability.	Lack of instructions and guidance form the healthcare facility
Jaswal. N et.al,2024 [37]	Age (40–60), Sex(females), Community Health centers > LTFU rate (compared to PHC’s & H&WC), Knowledge of the disease, To travel a long distance from their respective residences to the health centers	Poor dietary counseling	Medication side effects, Pill burden, Lifelong/long-term medication, Poor compliance to treatment.	DNS
Akinniyi. AA et. al, 2017 [41]	Forgot the appointment, Financial constraints for logistics, Conflicting commitments/Difficulty getting off work, Distance from the hospital, No one to escort me to the hospital (No family support), Strike actions/Public holidays	Discouraged by the long waiting time, Poor relationship with healthcare provider, Improper follow-up/Different physician seen at each visit, Dissatisfaction with the quality of healthcare provided.	Felt well/No new complaints/ Asymptomatic, Too ill to attend the appointments, Used alternative forms of treatment, No perceived benefit of treatment.	Poor relationship with healthcare provider, Perceived disrespect/negative attitude of healthcare provider.
Magadzire. BP et.al, 2017 [1]	Mobility and temporary migration, Forgetting or mixing up of appointments, Work commitments.	DNS	Switch to private medical care.	DNS
Mahmood. S et al, 2020 [39]	Age, Gender (Females), Marital status (Married), Lower level of education, Entitlement status(NO - free medical care/ Non- entitled), Irregular follow up	DNS	Treatment duration<5 years, Number of medications, Absence of co-morbid condition, Poor medication adherence, Low blood pressure control.	DNS
S.Devkota et al., 2016 [38]	Lack of awareness of hypertension, refusal to start medication, fear of side effects, low adherence, use of home remedies, stopping medication when asymptomatic	Long waiting times, poor counseling, inadequate communication, lack of consistent guidelines, weak primary-care follow-up systems	Monotherapy, uncontrolled blood pressure, medication non-adherence, stopping follow-up when symptoms subside, lack of lifestyle counseling	Communication gaps between patients and providers
Yirga GK et al.,2024 [42]	Absence of symptoms, poor awareness of hypertension complications, Age distribution (>50 yrs majority),	Long distance from health facility	Pill burden, poor knowledge of complications	Lack of reminder
Abbas H et.al, 2020 [43]	Older age (≥65 yrs), marital status (married),Obesity, Smoking cigarettes, Smoking hookah, Smoking both, Poor awareness about adherence, Lack of belief in treatment effectiveness, Not checking BP at home.	Health insurance reduced non-adherence (lack of insurance → higher non-adherence), Community pharmacies (40.2%), Tertiary hospital (33.3%), Private clinics (26.5%)	Uncontrolled BP associated with higher non-adherence, Pill interactions/side effects rarely reported, Self-reported non-adherence, Medication regimen adherence	Social and psychological factors affecting adherence, Stress control
Saguner A.M. et al, 2022 [45]	Older age, Female sex, Higher grades of obesity, Somatoform disorder, Depression (non-significant trend), Previous stroke	LTFU due to moving/leaving country	Hypertensive heart disease, Coronary artery disease, Thyroid disease, Hyperthyroidism, Higher number of antihypertensive drugs, Poor adherence (strongest factor)	Emotional stress reported as crisis trigger in some patients
Belay DG & Adugna A, 2022 [44]	Older age (implied for HIV, > 60 years), Fear of COVID-19 at hospital, financial constraints, transportation problems	Hospital overcrowding with COVID-19 patients, Mobility restrictions due to lockdowns, Medication stock outs`	Hypertension associated with severe COVID-19 risk, Use of ACE2-stimulating drugs in HTN/DM may increase COVID-19 severity	DNS

## Discussion

Hypertension is one of the leading causes of premature death, and a dangerous illness that raises the risk of serious issues of the heart, kidneys, brain, and other organs.

Two-thirds of cases of hypertension are found in LMIC, where the burden is disproportionately high. This is primarily because these populations have higher risk factors. The age-standardized prevalence of hypertension is higher in low- and middle-income countries than in high-income countries (HIC), at 31.5% and 28.5%, respectively [20]. According to the World Health Organization reports, the number of adults with hypertension illness has increased in the European, Americas, South-East Asia and Western Pacific areas. The increase is 42% in the European region and the Americas and 144% in the South East Asia and Western Pacific regions [46]. About one in five adults with hypertension (21%) have their blood pressure under control [20], with a national prevalence of 18.3%, and it was shown that men were more likely than women to have it [47]. The burden of vascular and renal disorders will increase as a result of these low identification and treatment rates combined with the rising number of people with hypertension [48]. Despite the availability of efficacious treatment, only a small percentage of adults with hypertension receive a diagnosis and the necessary recommended treatment.

One of the main causes of hypertension’s rising prevalence, particularly in LMIC, is the gap in its management. Missing follow-up raises the chance of health issues, which has a detrimental impact on quality of life by raising the risk of death, further health complications, and managing late effects from treatment non-follow-up [47]. LTFU patients can significantly impact healthcare efficiency, disrupt treatment plans, and reduce patient outcomes [7,8]. Inadequate follow-up can lead to disease progression, increased morbidity, and mortality, affecting patient care [3,4].

Specifically, this review examined the variables linked to LTFU in people with hypertension. Overall, the design, setting, sample size, and source of identifying LTFU were all inconsistent. The demographics and methodologies used in the various investigations varied significantly. All of the eligible hypertension patients were chosen as study participants from a large pool of hospitals, primary healthcare facilities, and communities using a majority of sequential and purposive sampling procedures. Several factors that affect regular follow-up were classified into patient, healthcare system, service & provider, treatment & disease, and interpersonal characteristics. Of these, the majority were specifically focused on patient factors. Many factors linked to LTFU of hypertension were consistently identified across countries. Likewise, there were differences in the operational definitions and terminology used to refer “LTFU.”

This study found a consistent correlation between LTFU and sociodemographic characteristics as age, sex, education, and work position. Findings from scoping research that looked into the parameters linked to LTFU in chronic diseases in high-income nations were inconsistent [4]. Patient characteristics related to clinic accessibility—such as transportation difficulties and long distances between the patient’s residence and the health facility—were consistently associated with higher LTFU. Limited clinic accessibility and transportation barriers had a clear negative effect on patients’ ability to return for scheduled care. These challenges were further compounded by drug stock-outs and reduced availability of services, particularly during periods of health system strain such as the COVID-19 pandemic [44]. Evidence from a systematic review aimed at reducing transportation barriers also showed that these factors significantly influenced treatment continuity and increased the overall cost of obtaining medications and care [49]. Other characteristics that were noted in our review included knowledge about the disease condition, lack of family support, and forgetting or missing and mixing appointments. Therefore, lowering transportation barriers (such as paying for transportation, setting up and connecting patients for transportation, and enabling self-management through remote monitoring and e-consultations) may be a useful strategy to lower the number of patients who being LTFU for treatment. Patients’ financial situation has a significant impact on whether they continue to seek medical care; our research revealed that individuals without insurance or experiencing financial hardships were more likely to be LTFU for treatment. Alemayehu et.al, in their study on out-of-pocket medical expenses among hypertensive patients, found that high out-of-pocket medical expenses are linked to both being uninsured and the type of health insurance coverage [50]. According to this review, LTFU was linked to longer wait times and lower levels of service satisfaction. Ferreira D.C et al in their systematic review reported that longer wait times result in significantly higher levels of dissatisfaction [51]. Our research also identified other issues, including poor patient-physician relationships, a lack of supervision and instructions, high service costs, inadequate counseling, and a different doctor seen at each session. Secondary research conducted by Derington C. et al. revealed that medication burden has minimal association with adherence and satisfaction of care provided, but it was significantly linked to patients not adhering to their treatment plans and failing to follow up [52]. An included study reported that COVID-19 led to global disruptions in hypertension services, resulting in reduced treatment continuity and higher LTFU rates and a significant drop in new hypertension treatment initiation [44]. Thus, altering behavior and increasing patient education and awareness could help lower LTFU rates, keep patients involved, and avoid financial losses. Patients with more severe conditions or those with co-morbid conditions may require numerous follow-up sessions to closely monitor the progress of their illness. However, the patient’s functional health status may be impacted by the negative correlations, such as increased disease severity, and their level of dissatisfaction may hinder their capacity to return for follow-up care.

However, the primary focus of this review was to identify factors associated with LTFU, few studies also highlighted potential factors which enhances patient compliance. Interventions such as patient education, improved access to healthcare facilities, consistent follow-up systems, effective communication between patients and healthcare providers, and streamlined service delivery were associated with better adherence. These findings suggest that strengthening health system responsiveness and patient engagement may play a key role in improving adherence and reducing LTFU, and should be further explored in future research.

In conclusion. the commonly identified factors for the hypertensive patients being LTFU reported in this review are 1) Sociodemographic factors; age; mainly the elderly populations, low education and income levels, no insurance coverage, cost related to transportation and medications, mobility and migration from the previous residence, far distance from the facility 2) Health awareness/ health information from healthcare professionals; lack of knowledge about the disease condition and its related co- morbidities, shifting for traditional medicines, 3) Perceptions and forgetfulness; no improvement in the symptoms, drug side effects, polypharmacy, absence of symptoms, pill burden, missing and forgetting the scheduled appointments 4) Service and medication requirements, service dissatisfaction, longer waiting time in physician clinics,. Because these comprehensive factors plays a pivotal role in patients not being LTFU for treatment.

### Limitations

This scoping review has several limitations. Only English-language studies were included, which may have led to the exclusion of relevant evidence. The focus on LTFU among hypertensive populations limits the generalizability of findings to other disease conditions. Although the included studies included diverse geographical settings, heterogeneity in study designs and reporting limited definitive conclusions about the impact of geographical patterns and factors on LTFU.

## Conclusion

This scoping review assessed the LTFU factors of hypertensive individuals. Numerous significant factors were identified, including patient demographics, healthcare services and providers, treatment, and interpersonal aspects. Nevertheless, LTFU was positively associated with financial limitations (no insurance, transportation), lack of access to care (distance between home and facility), ignorance of the disease, having several chronic conditions, being asymptomatic, pill burden or polypharmacy, competing commitments, misperceptions regarding the disease condition and longer clinic wait times that led to dissatisfaction. Globally, COVID-19 caused widespread interruptions to hypertension services, contributing to decreased treatment continuity and higher LTFU rates. Although the operational definitions and terminology used to characterize LTFU in healthcare settings differed significantly amongst research, we also found from the study that no-show patients in physician’s clinics were defined as LTFU for care if they were absent for six months or longer. Our findings indicated the importance of these four factor categories, which will provide a fundamental basis for future interventional and policy-related research on the prevalence and reduction of LTFU. Overall, the findings underscore the need for multifaceted strategies that address structural, behavioral, and service-delivery constraints. Strengthening patient education, improving medication availability, reducing transportation barriers, ensuring consistent follow-up systems, and enhancing communication between providers and patients are essential steps toward reducing LTFU and improving long-term hypertension management.

Future research should focus on qualitative studies to better understand the underlying reasons for LTFU, including the role of interpersonal factors in influencing follow-up behavior. Additionally, health literacy, as a key determinant of self-management in hypertension, warrants further exploration. Interventional studies aimed at improving follow-up engagement, along with strategies to strengthen health system responsiveness and patient engagement, may play a crucial role in enhancing adherence and reducing LTFU.

## Supporting information

S1 Table Data charting table: Comprehensive table which summarizes key characteristics of the included studies. (DOCX)

S2 Table Quality appraisal of included studies. Assessment of methodological quality of the included studies.
(DOCX)

S3 Table Data extraction sheet: Detailed extraction of relevant variables and information from all included studies. (XLSX)

S1 File Search strategy: Complete search strategies used across all databases for study identification. (DOCX)

S2 File PRISMA Checklist: Checklist outlining adherence to PRISMA guidelines for reporting the scoping review. From: Tricco AC, Lillie E, Zarin W, O’Brien KK, Colquhoun H, Levac D, et al. PRISMA Extension for Scoping Reviews (PRISMAScR): Checklist and Explanation. Ann Intern Med. 2018;169:467–473. https://doi.org/10.7326/M18-0850.
(DOCX)
