# Supplementary material for: Factors associated with lost to follow-up (LTFU) among patients with hypertension: A scoping review
Source: PLOS Glob Public Health. 2026 Jun 30;6(6):e0006240. doi: 10.1371/journal.pgph.0006240 (PMC13318019; doi:10.1371/journal.pgph.0006240)
Supplement: S1 Table — (DOCX) [file pgph.0006240.s001.docx]

| **Title of the study** | **Author/Country/**  **Year of publication** | **Factors associated with LTFU** | | | |
| --- | --- | --- | --- | --- | --- |
|  |  | **Patient characteristics** | **Healthcare system, service & provider characteristics** | **Treatment & Disease related characteristics** | **Interpersonal factors** |
| The impact of the COVID-19 pandemic on rates and predictors of missed hospital appointments in multiple outpatient clinics of The Royal Hospital, Sultanate of Oman: a retrospective study | Alawadhi. A et.al, Oman, 2023 | Sociodemographic: Sex, Age category, Nationality, Marital status, Type of Health insurance coverage, Distance from Patients residence to hospital. | Service cost, longer waiting times, and Appointment days. | DNS | DNS |
| Non- communicable disease care in Sierra Leone: a mixed- methods study of the drivers and barriers to retention in care for hypertension | Dibba. Y et.al, USA, 2024 | Age, Gender (Females), Health insurance, Transportation cost, Free medications/ Drug costs. | Long wait times, No frequent health education sessions/ talks. | Patients with blood pressure >180/110mmHg, Asymptomatic/ Absence of symptoms, Misunderstanding the long- term management of hypertension. | DNS |
| Non-communicable disease clinics in rural Ethiopia: why patients are lost to follow-up | Y. Mamo et.al, Ethiopia, 2019 | Age, Education level (illiterate), Socioeconomic status (low income level), Lack of knowledge, Cost of travel (by public transport/ walk) , Unable to stop working, Distance from home to healthcare, Drug costs, Treatment costs. | Service dissatisfaction, Drugs not available. | Much improved health (Absence of symptoms), No improvement in disease, Failure to cure, Drug side effects, Preferring other modes of treatment (use of traditional medicines/ treatment), Misunderstood about the treatment, Did not feel unwell during scheduled appointment/Asymptomatic | DNS |
| Factors affecting non-adherence to medical appointments among patients with hypertension at public health facilities in Punjab, India | Das. B et.al, Punjab, India , 2020 | Far distance from the facility, No time for a visit. | Lack of instructions/guidance from the facility, no proper instructions on when and where to come for a follow-up visits, long waiting time at the facilities, Poor quality of medications at public facilities, Medicines unavailability. | Lack of perceived illness, Acute health conditions/disability. | Lack of instructions and guidance form the healthcare facility |
| Factors affecting patient retention to hypertension treatment in a North Indian State: A mixed- method study | Jaswal. N et.al, Haryana, India, 2024 | Age (40-60), Sex(females), Community Health centers > LTFU rate (compared to PHC's & H&WC), Knowledge of the disease, To travel a long distance from their respective residences to the health centers | Poor dietary counseling | Medication side effects, Pill burden, Lifelong/long-term medication, Poor compliance to treatment. | DNS |
| Missed medical appointment among hypertensive and diabetic outpatients in a tertiary healthcare facility in Ibadan, Nigeria | Akinniyi. AA et. al, Nigeria, 2017 | Forgot the appointment, Financial constraints for logistics, Conflicting commitments/Difficulty getting off work, Distance from the hospital, No one to escort me to the hospital (No family support), Strike actions/Public holidays | Discouraged by the long waiting time, Poor relationship with healthcare provider, Improper follow-up/Different physician seen at each visit, Dissatisfaction with the quality of healthcare provided. | Felt well/No new complaints/ Asymptomatic, too ill to attend the appointments, Used alternative forms of treatment, No perceived benefit of treatment. | Poor relationship with healthcare provider, Perceived disrespect/negative attitude of healthcare provider. |
| Association between attendance at outpatient follow-up appointments and blood pressure control among patients with hypertension | Magadzire. BP et.al, South Africa, 2017 | Mobility and temporary migration, Forgetting or mixing up of appointments, Work commitments. | DNS | Switch to private medical care. | DNS |
| Reasons for missed appointments linked to a public-sector intervention targeting patients with stable chronic conditions in South Africa: results from in-depth interviews and a retrospective review of medical records | Mahmood. S et al, Pakistan, 2020 | Age, Gender (Females), Marital status (Married), Lower level of education, Entitlement status(NO - free medical care/ Non- entitled), Irregular follow up | DNS | Treatment duration<5 years, Number of medications, Absence of co-morbid condition, Poor medication adherence, Low blood pressure control. | DNS |
| Non-adherence to appointment follow-up and its associated factors among hypertensive patients in follow-up clinics in South Gondar hospitals | Gebrie Kassaw Yirga et al. Ethiopia 2024 | Age, Gender(females), marital status ((Married), Lower level of education (Unable to read & write / Primary school), Entitlement status (No – Not insured / Non-entitled), Irregular follow-up (non-adherent: missed >3 out of 10 appointments), Absence of perceived symptoms (patients felt “healthy”)  • Poor awareness about hypertension complications |  |  | Lack of reminders for follow-up, Poor provider communication (inadequate instructions given) |
| Barriers to Treatment and Control of Hypertension Among Hypertensive Participants: A Community-Based Cross-Sectional Mixed-Method Study in Municipalities of Kathmandu, Nepal | S. Devkota et al., Kathmandu, Nepal, 2016 | Lack of awareness of hypertension, refusal to start medication, fear of side effects, low adherence, use of home remedies, stopping medication when asymptomatic | Long waiting times, poor counseling, inadequate communication, lack of consistent guidelines, weak primary-care follow-up systems | Monotherapy, uncontrolled blood pressure, medication non-adherence, stopping follow-up when symptoms subside, lack of lifestyle counseling | Communication gaps between patients and providers |
| Non-adherence to appointment follow-up and its associated factors among hypertensive patients in follow-up clinics in South Gondar hospitals | Yirga GK et al., South Gondar Zone, Ethiopia, 2024 | Absence of symptoms, poor awareness of hypertension complications, Age distribution (>50 yrs majority), | Long distance from health facility | Pill burden, poor knowledge of complications | Lack of reminder |
| Factors Associated with Antihypertensive Medication Non-Adherence: A Cross-Sectional Study Among Lebanese Hypertensive Adults | Abbas H et.al, Lebanon,2020 | Older age (≥65 yrs), marital status (married),Obesity, Smoking cigarettes, Smoking hookah, Smoking both, Poor awareness about adherence, Lack of belief in treatment effectiveness, Not checking BP at home. | Health insurance reduced non-adherence (lack of insurance → higher non-adherence), Community pharmacies (40.2%), Tertiary hospital (33.3%), Private clinics (26.5%) | Uncontrolled BP associated with higher non-adherence, Pill interactions/side effects rarely reported, Self-reported non-adherence, Medication regimen adherence | Social and psychological factors affecting adherence, Stress control |
| Risk Factors Promoting Hypertensive Crises: Evidence From a Longitudinal Study | Saguner A.M. et al., Bern, Switzerland, 2010 | Older age, Female sex, Higher grades of obesity, Somatoform disorder, Depression (non-significant trend), Previous stroke | LTFU due to moving/leaving country | Hypertensive heart disease, Coronary artery disease, Thyroid disease, Hyperthyroidism, Higher number of antihypertensive drugs, Poor adherence (strongest factor) | Emotional stress reported as crisis trigger in some patients |
| Lost to follow-up from chronic care services during COVID-19 from health facilities, Northwest Ethiopia | Belay DG & Adugna A, Northwest Ethiopia (Gondar City), 2022 | Older age (implied for HIV, >60 years), Fear of COVID-19 at hospital, financial constraints, transportation problems | Hospital overcrowding with COVID-19 patients, Mobility restrictions due to lockdowns, Medication stock outs` | Hypertension associated with severe COVID-19 risk, Use of ACE2-stimulating drugs in HTN/DM may increase COVID-19 severity | DNS |
