# Supplementary material for: Factors associated with lost to follow-up (LTFU) among patients with hypertension: A scoping review
Source: PLOS Glob Public Health. 2026 Jun 30;6(6):e0006240. doi: 10.1371/journal.pgph.0006240 (PMC13318019; doi:10.1371/journal.pgph.0006240)
Supplement: S2 Table — Assessment of methodological quality of the included studies. (DOCX) [file pgph.0006240.s002.docx]

**CROSS – SECTIONAL STUDY:**

| CROSS- SECTIONAL STUDY (JBI Critical Appraisal tool) | | | | | | |
| --- | --- | --- | --- | --- | --- | --- |
| ITEMS | Y. Mamo,  2019 | Akinniyi. AA,2017 | Mahmood. S,2020 | H.Abbas,  2020 | D.GBelay,  2022 | YirgaG.K,  2024 |
| Q1 | 2 | 1 | 2 | 2 | 2 | 2 |
| Q2 | 2 | 2 | 2 | 2 | 2 | 2 |
| Q3 | 1 | 0 | 2 | 2 | 2 | 1 |
| Q4 | 2 | 2 | 2 | 2 | 2 | 2 |
| Q5 | 0 | 0 | 0 | 2 | 1 | 2 |
| Q6 | 0 | 0 | 0 | 2 | 0 | 2 |
| Q7 | 0 | 2 | 0 | 2 | 2 | 0 |
| Q8 | 1 | 2 | 2 | 2 | 1 | 2 |
| SCORE | \| 50% \| \| --- \| | \| 56.25% \| \| --- \| | \| 62.5% \| \| --- \| | \| 100% \| \| --- \| | \| 75% \| \| --- \| | \| 81.25% \| \| --- \| |
| QUALITY | Moderate Quality | Moderate Quality | Moderate Quality | High Quality | High Quality | High Quality |

**QUALITATIVE STUDY:**

| QUALITATIVE STUDY (JBI Critical Appraisal tool) | |
| --- | --- |
| ITEMS | Das. B, 2020 |
| Q1 | 0 |
| Q2 | 2 |
| Q3 | 2 |
| Q4 | 2 |
| Q5 | 2 |
| Q6 | 2 |
| Q7 | 2 |
| Q8 | 2 |
| Q9 | 2 |
| Q10 | 0 |
| SCORE | 80% |
| QUALITY | High Quality |

**MIXED – METHOD STUDY:**

| Mixed-Methods Study (MMAT 2018) | | | | |
| --- | --- | --- | --- | --- |
| ITEMS | Dibba Y., 2024 | Jaswal N., 2024 | Magadzire BP., 2017 | S. Devkota, 2016 |
| S1. | 2 | 2 | 2 | 2 |
| S2. | 2 | 2 | 2 | 2 |
| Q1. 1.1 | 2 | 2 | 2 | 2 |
| Q1. 1.2 | 2 | 2 | 2 | 2 |
| Q1. 1.3 | 2 | 2 | 2 | 2 |
| Q1. 1.4 | 2 | 2 | 2 | 2 |
| Q1. 1.5 | 2 | 2 | 2 | 2 |
| Q4. 4.1 | 2 | 2 | 2 | 2 |
| Q4. 4.2 | 2 | 2 | 2 | 1 |
| Q4. 4.3 | 2 | 2 | 2 | 2 |
| Q4. 4.4 | 1 | 1 | 1 | 1 |
| Q4. 4.5 | 2 | 2 | 2 | 2 |
| Q5. 5.1 | 1 | 0 | 0 | 2 |
| Q5. 5.2 | 2 | 2 | 2 | 2 |
| Q5. 5.3 | 2 | 2 | 1 | 2 |
| Q5. 5.4 | 0 | 0 | 0 | 2 |
| Q5. 5.5 | 2 | 2 | 2 | 2 |
| Score | 88.24% | 85.29% | 82.35% | 94.12% |
| Quality | High Quality | High Quality | High Quality | High Quality |

**RETROSPECTIVE CASE CONTROL:**

| RETROSPECTIVE CASE CONTROL (NOS Scale- Case control studies) | |
| --- | --- |
| ITEMS | Alawadhi. A, 2023 |
| Q1 | 2 |
| Q2 | 2 |
| Q3 | 1 |
| Q4 | 2 |
| Q5 | 2 |
| Q6 | 0 |
| Q7 | 2 |
| Q8 | 2 |
| Q9 | 0 |
| SCORE | 6 stars |
| QUALITY | Moderate Quality |

**COHORT STUDY:**

| COHORT STUDY: | |
| --- | --- |
| ITEMS | Saguner et. al |
| Q1 | 2 |
| Q2 | 2 |
| Q3 | 2 |
| Q4 | 2 |
| Q5 | 2 |
| Q6 | 2 |
| Q7 | 2 |
| Q8 | 2 |
| Q9 | 2 |
| SCORE | 100% |
| QUALITY | High quality |
