# Supplementary material for: Factors associated with lost to follow-up (LTFU) among patients with hypertension: A scoping review
Source: PLOS Glob Public Health. 2026 Jun 30;6(6):e0006240. doi: 10.1371/journal.pgph.0006240 (PMC13318019; doi:10.1371/journal.pgph.0006240)
Supplement: S1 File — (DOCX) [file pgph.0006240.s004.docx]

**SEARCH STRATEGY:**

| Databases | Search strategy | No. of hits |
| --- | --- | --- |
| PubMed  Searched on:  09/12/2025 | (((("Lost to Follow-Up"[Mesh]) OR ("No-Show Patients"[Mesh])) OR ("Patient Dropouts"[Mesh])) OR (missed visit*)) AND ((((((hypertension [All fields]) OR (hypertensive [All fields])) OR (hyperten*[All fields])) OR (high blood pressure*[All fields])) OR (elevated blood pressure*[All fields])) OR (abnormal blood pressure*[All fields])) | 386 |
| Scopus  Searched on:  09/12/2025 | "Hypertension" OR "hypertensive" OR "hyperten*" OR "high blood pressure*" OR "elevated blood pressure*" OR "abnormal blood pressure*" AND "Lost to follow up" OR "No-show patient*" OR "Patient dropout*" OR "missed visit*" AND PUBYEAR > 2009 AND PUBYEAR < 2026 | 2554 |
| Ovid Medline  Searched on:  09/12/2025 | (Hypertension or hypertensive or hyperten* or "high blood pressure*" or "elevated blood pressure*" or "abnormal blood pressure*").mp. AND ("Lost to follow up" or "No-show patient*" or "Patient dropout*" or "missed visit*").mp. | 612 |
| WOS:  Searched on:  09/12/2025 | (((("Lost to Follow-Up") OR ("No-Show Patients")) OR ("Patient Dropouts")) OR ("missed visit*" )) AND ((((((hypertension) OR (hypertensive)) OR (hyperten*)) OR ("high blood pressure*")) OR ("elevated blood pressure*")) OR ("abnormal blood pressure*"))  <https://www.webofscience.com/wos/woscc/summary/1e531ad8-e488-477d-b56a-8c3489ef0065-018fbb6ecb/relevance/1> | 440 |
